# Fecal Microbiota Transplantation in Refractory Immune-Mediated Colitis: Case Series and Review of the Literature

**DOI:** 10.3390/ph18111719

**Published:** 2025-11-12

**Authors:** Marin Golčić, Laura Radoš, Iva Skočilić, Ivona Badovinac, Goran Hauser, Irena Krznarić Zrnić, Marina Šantić, Dora Fučkar Čupić, Sara Francetić, Karla Lisica, Lea Juras, Marija Škrtić, Ana Bešvir Džubur, Robert Šeparović, Vedran Tomašić, Ana Tečić Vuger, Ivana Mikolašević

**Affiliations:** 1 Clinic for Tumors, Clinical Hospital Centre Rijeka, 51000 Rijeka, Croatia; marin.golcic@gmail.com (M.G.); laura1301rados@gmail.com (L.R.); iskocilic@gmail.com (I.S.); ivona.badovinac@gmail.com (I.B.); sara.francetic3@gmail.com (S.F.); karla.lisica1@gmail.com (K.L.); juraslea32@gmail.com (L.J.); marijaskrtic96@gmail.com (M.Š.); besvirdzuburana@gmail.com (A.B.D.); 2 Department of Oncology and Radiotherapy, Faculty of Medicine, University of Rijeka, 51000 Rijeka, Croatia; 3 Department of Gastroenterology, Clinical Hospital Centre Rijeka, 51000 Rijeka, Croatia; goran.hauser@medri.uniri.hr (G.H.); ikrznariczrnic@yahoo.co.uk (I.K.Z.); 4 Department of Internal Medicine, Faculty of Medicine, University of Rijeka, 51000 Rijeka, Croatia; 5 Institute of Microbiology and Parasitology, Faculty of Medicine, University of Rijeka, 51000 Rijeka, Croatia; marina.santic@medri.uniri.hr; 6 Clinical Department of Pathology and Cytology, Clinical Hospital Centre Rijeka, 51000 Rijeka, Croatia; dorica9@gmail.com; 7 Department of General Pathology and Pathological Anatomy, Faculty of Medicine, University of Rijeka, 51000 Rijeka, Croatia; 8 Division for Medical Oncology, University Hospital for Tumors, Sisters of Mercy University Hospital Center, 10000 Zagreb, Croatia; robertseparov@gmail.com (R.Š.); ana.tecic@yahoo.com (A.T.V.); 9 Department of Gastroenterology and Hepatology, Clinical Hospital Center Sisters of Mercy, 10000 Zagreb, Croatia; tomasicvedran@gmail.com

**Keywords:** immune-mediated colitis, immune checkpoint inhibitors, corticosteroids, infliximab, fecal microbiota transplantation

## Abstract

**Background/Objectives:** Immune checkpoint inhibitors (ICI) represent a significant breakthrough in cancer management, but they can cause adverse effects such as immune-mediated colitis (IMC). The standard first-line treatment is corticosteroids, and second-line treatment is infliximab or vedolizumab. However, a proportion of immune-mediated colitis (IMC) cases are refractory to immunosuppressive treatment, which has led to the exploration of novel approaches such as fecal microbiota transplantation. **Methods:** We present two patients who both developed grade III IMC following application of durvalumab and pembrolizumab, respectively. Both patients were refractory to corticosteroid therapy, while the first one also showed no improvement to infliximab. We performed two separate applications of FMT on both patients, from different donors, as a rescue treatment. **Results:** After unsuccessful immunosuppressive treatment and following rescue FMT, both patients demonstrated a rapid and sustained improvement in inflammatory markers, clinical symptoms, quality-of-life scores, and colonoscopy findings, without additional immunosuppressive treatment. **Conclusions:** FMT appears to be safe and a potentially effective treatment option for patients with refractory IMC both as second- and third-line therapy options. Continued efforts toward rigorous donor screening, use of standardized biobanks, and standardizing FMT protocols will further enhance safety and reproducibility.

## 1. Introduction

Immune checkpoint inhibitors ( ICIs) represent a breakthrough in cancer treatment, providing dramatic improvements in survival across various cancers, including non-small cell lung cancer (NSCLC) [1]. ICIs block key interactions between cancer cells and checkpoint molecules such as cytotoxic T-lymphocyte-associated protein 4 (CTLA-4, e.g., ipilimumab), programmed cell death 1 (PD-1, e.g., nivolumab), and programmed cell death ligand 1 (PD-L1, e.g., atezolizumab). These molecules are often upregulated by cancers to suppress T-cell function within the tumor microenvironment. By blocking these checkpoints, ICIs restore T-cell activation and proliferation, enhance cytotoxic activity, and promote a more effective antitumor response [2,3,4]. However, ICI therapy can lead to severe immune-related adverse events affecting various organs [5]. Immune-mediated colitis (IMC) is an immune-mediated inflammatory bowel condition that arises due to systemic activation of T lymphocytes and cytokines, combined with dysbiosis of the intestinal microbiota, leading to autoimmune-type inflammation of the intestinal mucosa [6]. The incidence of IMC is up to 32%, with grade ≥3 events occurring in up to 21% of patients [5].

The treatment of IMC depends on severity. Mild cases may require only symptomatic therapy, while more severe cases are managed with corticosteroids as first-line treatment, followed by infliximab or vedolizumab for refractory disease [7,8]. Up to 41% of patients with grade ≥2 IMC are refractory to corticosteroids but respond to infliximab, while approximately 11% are refractory to both corticosteroids and infliximab [8]. Hence, there is a need to improve the therapeutic outcomes of those patients. Recent data have implicated the gut microbiome as an important factor in IMC development, and tools that modulate the microbiome could potentially lead to improvement of the condition [9].

Fecal microbiota transplantation (FMT) has emerged as a potential treatment for refractory IMC, with a growing number of reports documenting its effectiveness, although there is no gold standard regarding the donor choice or number of FMT applications [10,11,12,13,14]. FMT involves the administration of a fecal suspension from a healthy donor into the gastrointestinal tract of a recipient, usually via endoscopy, with the aim of restoring or enriching the recipient’s intestinal microbiota. Current guidelines recommend FMT in immunocompetent adults with recurrent *Clostridioides difficile* infection following standard-of-care antibiotics [15]. Emerging evidence also supports its role in other gastrointestinal conditions [16] and even as a potential anticancer tool [17,18,19,20,21]. Here, we present two cases: a 75-year-old female patient with grade III IMC refractory to corticosteroids and infliximab and a 50-year-old male patient with grade III IMC refractory to corticosteroids, both of whom experienced clinical benefit from FMT.

## 2. Case Presentations

The first patient was diagnosed with stage IIIA lung adenocarcinoma in February 2024. The tumor was negative for epidermal growth factor receptor (EGFR), anaplastic lymphoma kinase (ALK), ROS proto-oncogene 1 (ROS1) mutations and demonstrated a programmed death-ligand 1 (PD-L1) expression of 60%. Following discussion at a multidisciplinary team (MDT) meeting, concomitant chemoradiotherapy with durvalumab maintenance was recommended. Prior to her cancer diagnosis, the patient had several comorbidities, including hypothyroidism, chronic heart failure, paroxysmal atrial fibrillation, and chronic kidney disease. However, her Eastern Cooperative Oncology Group (ECOG) performance status was 1, and she was deemed fit for treatment.

**Figure 1:**
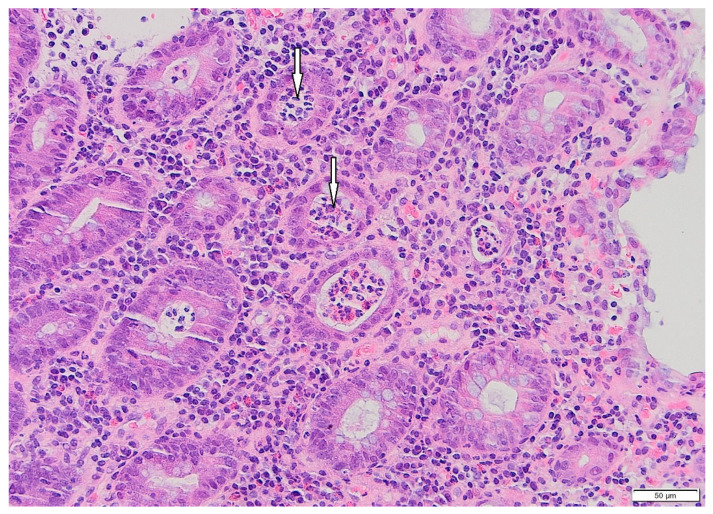
Patient#1. Histopathological view of the fragment of colonic mucosa (HE, ×200). Active colitis, with neutrophilic crypt microabscesses (arrows), is evident.

**Figure 2:**
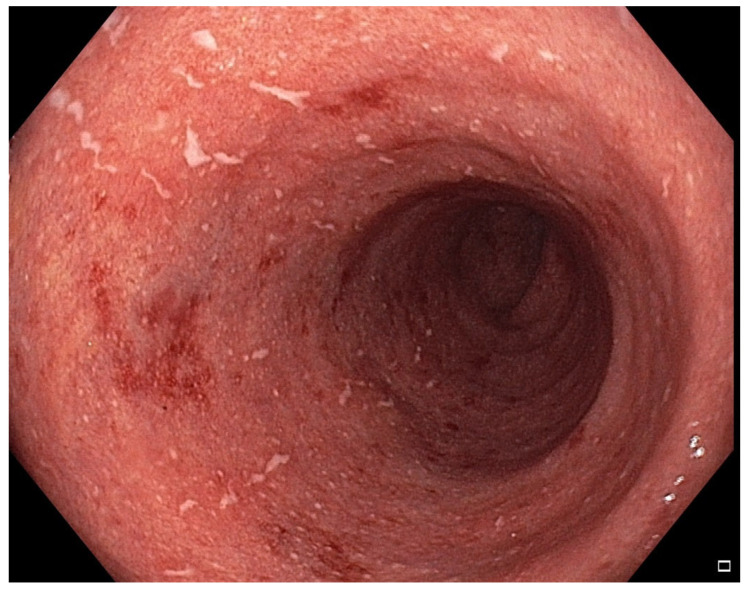
Patient#1. Inflammatory mucosal changes with ulcers and bleeding consistent with immune-mediated colitis demonstrated during the first colonoscopy.

From March to June 2024, the patient received four cycles of cisplatin and pemetrexed, followed by external beam radiotherapy (6600 cGy in 33 fractions) between August and October 2024. The patient tolerated initial treatment well, without significant adverse effects. Imaging demonstrated tumor shrinkage, and durvalumab (1500 mg every three weeks) was initiated in December 2024. After the third cycle in January 2025, the patient developed grade I IMC, which was treated conservatively. By February 2025, the symptoms progressed to increased bowel frequency (3–4 per day) with intermittent hematochezia. Laboratory and microbiological tests, including coagulation studies and stool cultures, were unremarkable. Colonoscopy and histopathological examination confirmed inflammatory mucosal changes consistent with IMC (Figure 1 and Figure 2).

The patient started prednisolone 1 mg/kg and initially clinically improved, allowing for steroid tapering. In April 2025, she developed pneumonia treated with levofloxacin, which was complicated by acute kidney injury. Upon tapering prednisolone to 5 mg daily, the colitis symptoms recurred. She was hospitalized in May 2025 with up to 10 bowel movements per day, abdominal pain, and hematochezia. Laboratory tests revealed a mildly elevated C-reactive protein (CRP) 12.2 mg/L and fecal calprotectin 3483 μg/g. No infectious pathogens were identified.

Despite intravenous corticosteroids and three doses of infliximab (completed by July 2025), symptoms worsened, with CRP 94.8 mg/L and calprotectin 30,080 μg/g. MDT consensus was to proceed with FMT using stool from the fecal biobank at the University of Rijeka School of Medicine, the only such biobank in the region. Colonoscopy revealed pancolitic inflammatory changes with ulcers and bleeding (Figure 2), and FMT was performed in the next step via the same colonoscopy.

The procedure involved preparing the patient with large-bowel cleansing according to a standard protocol. Stool from a healthy donor—previously screened for infectious diseases and other exclusion criteria in line with current guidelines (details available in the Supplement)—was then processed with saline solution at a ratio of 1:3–5. The homogenized suspension (300 mL) was thawed on the day of transfer and instilled into the recipient’s colon during colonoscopy. Although FMT can also be administered via a nasogastric, nasoduodenal, or nasojejunal tube, and these methods can even be combined, colonoscopy was our preferred approach, as it additionally allowed for direct evaluation of the colon. Additional details about the procedure and donor selection criteria are given in the Supplemental File.

**Figure 3:**
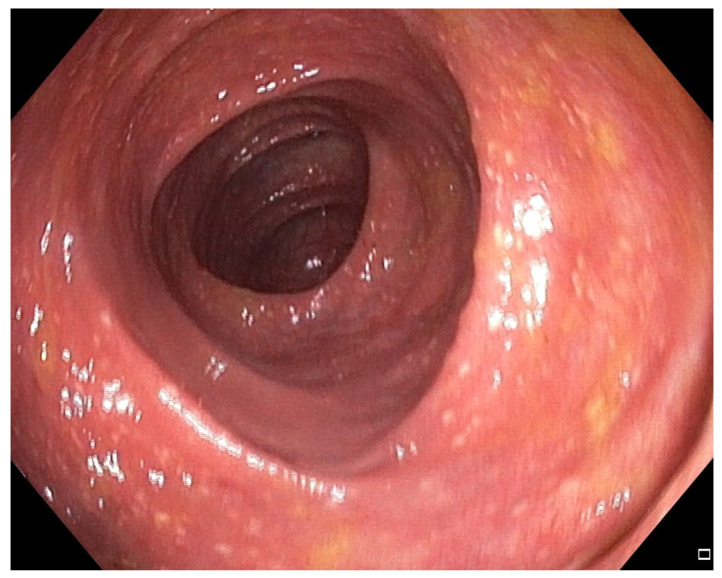
Patient#1. Mild inflammatory mucosal changes during the second colonoscopy.

**Figure 4:**
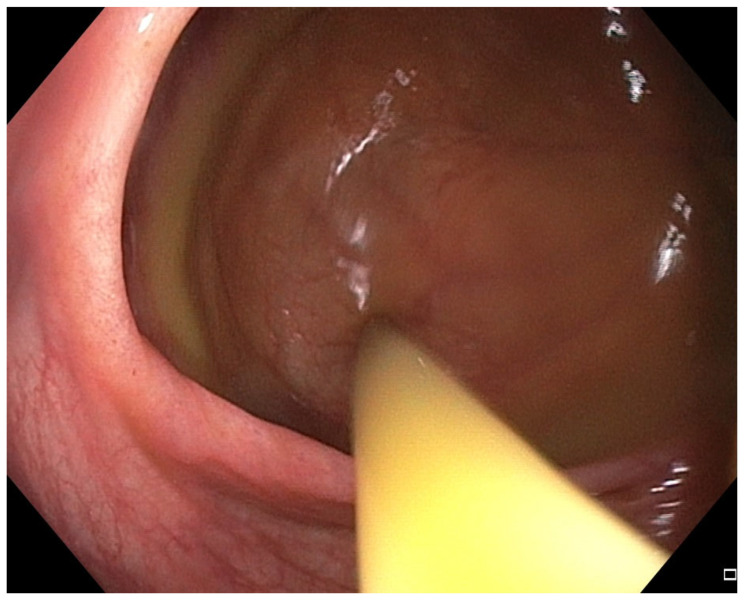
Patient#1. The application of fecal microbiota transplantation via colonoscopy.

The patient experienced rapid symptomatic improvement, but two days later developed pneumonia requiring antibiotics (levofloxacin). Computed tomography (CT) angiography excluded pulmonary embolism and demonstrated no progression of cancer. Antibiotic therapy resolved pneumonia but worsened colitis symptoms, although quantitative microbiome analysis of the drug’s impact on intestinal microbiota could not be performed due to unavailability of microbiome testing. A second FMT was performed 15 days after the first, using stool from a different donor. Colonoscopy revealed significantly less inflammation without ulcers and bleeding compared with the previous procedure (Figure 3 and Figure 4). The patient improved clinically and was discharged with IMC downgraded to grade I. Before discharge, CRP was 150.0 mg/L and calprotectin 7328 μg/g and one month later, CRP was 20.2 mg/L and calprotectin 1782 μg/g. Additionally, intestinal ultrasound was performed, demonstrating that the colon wall was thickened in the sigmoid part (ranging from 3.8 to 4.8 mm); the colon wall showed partially preserved stratification although in places the stratification was not visible, instead showing a homogenous hypoechoic wall. Around the inflamed segments, hypertrophic mesenteric fat tissue was present, but no enlarged lymph node or ascites were seen. Other parts of colon show no signs of inflammation. However, despite partial resolution on intestinal ultrasound, the patient remains off anticancer or immunosuppressive therapy, clinically stable on follow-up and with no disease progression on follow-up CT in September 2025.

The second patient was a 50-year-old man initially treated at another institution for melanoma of the parietal region. The initial diagnosis was made in December 2022, and confirmed superficially spreading melanoma pT1b, which was later upgraded to pT2b N1 after additional surgery and sentinel lymph node biopsy. The tumor was B-Raf proto-oncogene (BRAF) mutated. Due to local spreading and lack of available adjuvant therapy at the time, in February 2023, superficial parotidectomy and radical neck dissection were performed with additional one lymph node positive on histopathological finding. The patient was regularly followed-up and on positron emission tomography (PET)/CT in April 2025 a newly diagnosed solitary node was found on lower left lung with positive pathological metabolism, with multiple micronodules, metabolically negative but were newly registered. Additionally, a node was registered in left anterior abdominal wall.

**Figure 5:**
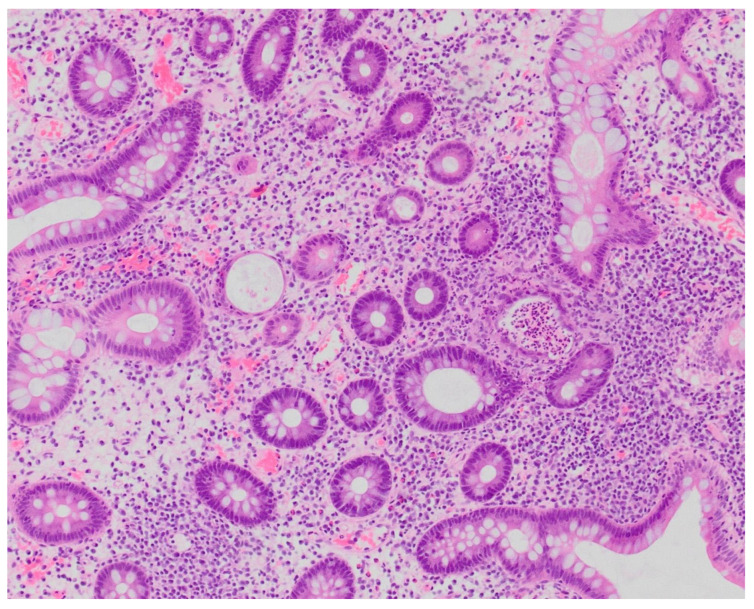
Patient#2. Histopathological view of the fragment of colonic mucosa demonstrating signs of chronic inflammation suggestive of immune-mediated colitis (×200).

Following oncological examination, the MDT recommended treatment with stereotactic body radiation therapy (SBRT) along with pembrolizumab. The patient started in May 2025, and the first radiological control demonstrated stable disease, without progression. However, in early August 2025 the patient developed significant diarrhea, which was classified as Grade 3 on Common Terminology Criteria for Adverse Events (CTCAE), along with fatigue. His adrenocorticotropic hormone (ACTH), cortisol, thyroid-stimulating hormone (TSH) and fT4 were all normal, although his CRP was 192 mg/L. Microbiology evaluation, including *C. difficile* (immunochromatographic method), was not positive for infectious agents. He was hospitalized and initially treated with i.v. corticosteroids (2 mg/kg) along with dual parenteral antibiotics (piperacillin/tazobactam and vancomycin). A colonoscopy was performed on 19th of August, revealing continuous mucosal inflammation from caecum to rectum, with multiple (average size 10–20 mm), shallow to deep confluent ulcers, without proliferative/stenosing changes. Histopathology confirmed changes consistent with IMC (Figure 5).

**Figure 6:**
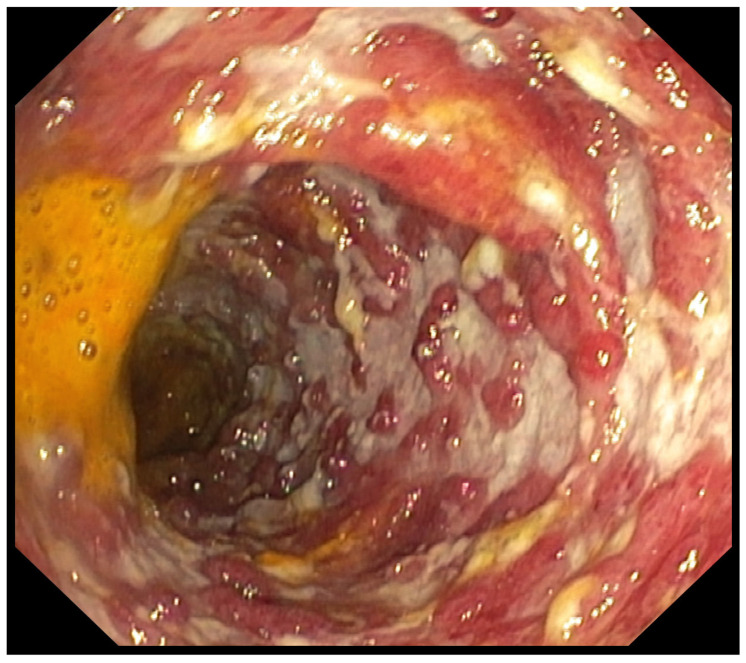
Patient#2. The initial colonoscopy confirmed significant ulcerations and inflammation of whole colon.

He was admitted to our institution on 27th of August 2025 as his clinical condition did not improve on initial corticosteroid treatment. The initial FMT was performed on the 29th with instillation with 300 mL of healthy donor stool; the colonoscopy confirmed significant ulcerations and inflammation of whole colon (Figure 6) and in the same act, the FMT was performed. We used available fecal material at previously mentioned biobank at Faculty of Medicine, University of Rijeka with donor criteria given in a Supplementary File.

**Figure 7:**
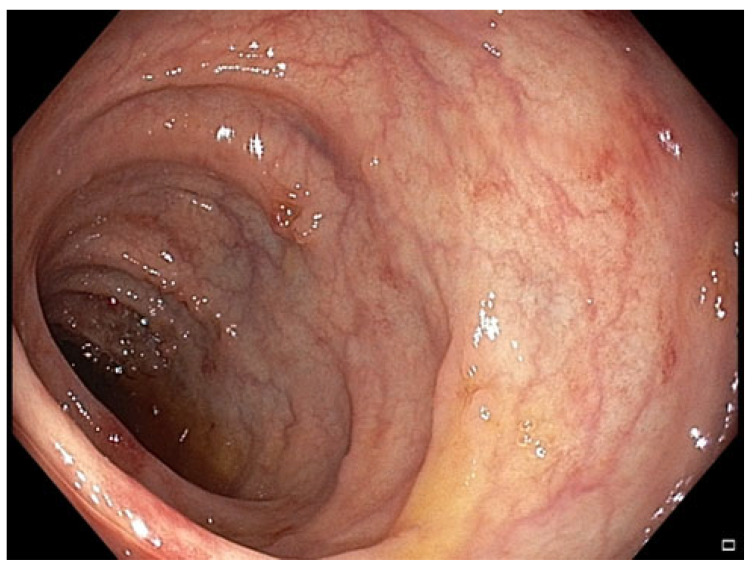
Patient#2. The control colonoscopy following the second FMT.

At that point, patient’s laboratory values were unremarkable except for leukocytosis (12.4 × 10^9^) and a CRP of 75.9 mg/L. The patient was also febrile up to 38.1°, but responsive to acetaminophen. The patient demonstrated rapid clinical improvement and had no fever the next day, with a single unformed stool the next day; the first formed, blood-free stool was registered 2 days following the FMT. Another FMT was performed with instillation of 300 mL of another healthy donor stool on 10th of September. The colonoscopy also demonstrated dramatic improvement of inflammation and ulcerations in the whole colon (Figure 7). The patient was discharged on 11th of September with a reduction in CRP to 13.5 mg/L with dramatic clinical improvement. Calprotectin after 1st FMT was 2411 µg/g, and 1539 µg/g on discharge. No corticosteroids or antibiotics were applied following FMT. The patient is currently undergoing a second-line therapy with dabrafenib/trametinib.

Both patients were evaluated using European Organisation for Research and Treatment of Cancer QoL quality of life questionnaire core-30 (EORTC QLQ-C30) [22], evaluating symptoms for the week before and 1 week after FMT. The permission to publish data with EORTC QLQ-C30 was given by EORTC and the results were given in Table 1, demonstrating a rapid and clinically meaningful improvement in the majority of symptoms.

While a longer follow-up is needed to confirm efficacy sustainability and long-term safety, at the time of writing, both patients remain clinically well more than 12 and 8 weeks, respectively.

## 3. Discussion

Despite the success of ICIs in cancer treatment, their use can lead to serious adverse effects, including IMC, with some patients being refractory to first- and second-line treatments. While data is limited, research suggests that the gut microbiome significantly influences the success of ICIs as well as the development of ICI adverse effects [23,24]. The potential modulation of the microbiota may be a therapeutic factor in IMC, as the microbiota plays a key role in gut health via immune response modulation, tumor microenvironment remodeling, and production of key metabolites [25]. Since it represents a form of microbiota modulation, FMT is an increasingly explored treatment method, not only because of its antitumor potential but also because of its role in the symptomatic treatment of IMC [10,11,12,13,17,18,19,20].

To the best of our knowledge, only a few reports evaluated the use of FMT as a third-line IMC treatment. The first study on this subject was published in 2018 by Wang et al. [10], who reported on two patients who both had successful third-line FMT. The first patient had unsuccessful corticosteroids and infliximab treatment and achieved complete response after a single FMT, while the second patient was refractory to corticosteroids, infliximab, and vedolizumab. This patient did not respond to the first FMT but achieved complete response after the second FMT. Interestingly, both patients had the same stool donor. They also studied the differences in microbiota composition before and after FMT, which showed alignment to the donor’s microbiota, but the results between the two patients were heterogeneous [10].

The second report on this topic was by Fasanello et al., who published a case report on a single patient who had a successful one-course FMT after failure of corticosteroids, infliximab, mycophenolate mofetil, mesalamine, and vedolizumab [11]. The third and largest report was published by Halsey et al. Their report on 12 patients offered a broader perspective on FMT success in IMC treatment. Out of 12 patients, 10 benefited from FMT, with 7 achieving complete response. Of the three patients who did not respond to the first FMT, one responded after a repeat FMT while two remained non-responders after two FMTs. They used four different healthy donors, and the repeat FMTs were from different donors. They also analyzed the microbiota before and after FMT. Responders showed a significant increase in alpha-diversity, an increase in genera such as Collinsella and Bifidobacterium, and alignment of recipient microbiota to donors [12]. Wang et al. recently reported preliminary results of a larger-scale study on 62 cancer patients with IMC receiving third-line FMT. Around 80% of patients benefited from FMT, while complications occurred in about 37% of patients. These results are promising and once fully published, may represent the most important contribution to the literature on this topic to date [13].

Another important contribution to the field was published by Elkrief et al., who demonstrated clinical improvement with FMT in five patients with IMC refractory to steroids and biologic anti-inflammatory agents [26]. While most research included patients receiving FMT following both corticosteroids and biological agents, Shatila et al. also included several patients who received FMT in earlier lines, with significant success [27]. While there is no robust data comparing FMT to standard second-line vedolizumab or infliximab in cancer patients with ICM, data from ulcerative colitis patients demonstrate that FMT is as efficacious and as safe as other targeted therapies, including infliximab [28]. Furthermore, for immunosuppressant-refractive colitis grades 3 or 4, National Comprehensive Cancer Network (NCCN) guidelines suggest that FMT may be considered based on institutional availability [7].

Unlike some of the previous trials, we used two applications of FMT with around 10-day difference in between both, with no antibiotic preparation. While one of our patients had a dramatic improvement after the first one, another patient required a second FMT, potentially due to antibiotic use after the first. We also used different donors for the two FMTs, unlike Wang et al. [10], who used the same donor stool and also achieved IMC remission. Halsey’s non-responders and partial responders received two FMT applications, after which one achieved complete response and two did not; however, they did not attempt a third FMT [12]. None of the existing reports mentioned the use of antibiotics at any point, unlike our first patient, who had to receive levofloxacin for pneumonia following the first FMT. This offers a unique perspective of FMT being successful even after further dysbiosis caused by antibiotic use following the initial FMT. While both infliximab and vedolizumab are acceptable options in corticosteroid-refractory setting, we used infliximab due to a lower recurrence rate of colitis [7,29]. Our results also demonstrate the feasibility of FMT in refractory IMC patients as both second- and third-line therapy, also showing that the speed of response varies in different patients despite using the same method. This could be due to patient related factors or the specifics of donor stool used.

Despite the promising results so far, there are still some risks associated with FMT. While adverse effects are usually low-grade, such as diarrhea, flatulence, bloating, mild abdominal pain, and low-grade fever, there remains a theoretical risk of serious toxicity in immunocompromised patients [30]. Furthermore, several practical questions remain unanswered. We are still unsure of the ideal stool donor type or ideal microbiota composition. There is also a discrepancy regarding the number of donor stool applications and whether different or the same donors should be used. While some patients in existing studies experienced IMC remission after a single FMT, others required multiple applications, and some did not respond at all. Currently, there is no fixed number of FMT applications recommended, though no more than two FMTs have been performed in studies so far. Existing reports also do not mention any special preparation for FMT other than standard colonoscopy preparation, and antibiotics are not mentioned except in this case report.

While the limitation of our case series is that we only reported on two cases, which could limit conclusion generalization, and did not use routine microbiome analysis, we did demonstrate the potential to use healthy donors from fecal biobank, following rigorous screening. Furthermore, the rapid effectiveness of the therapy was clinically shown through improvement in EORTC QLQ C30 scores. FMT is an inexpensive and clinically effective method that potentially be useful as a corticosteroid-sparing therapy, although large-sample studies and longer follow-up are needed to confirm our results.

Our data, along with other research on this topic [10,11,12,13,26,27] suggests that FMT may be considered as a third-line “rescue” option after unsuccessful 1st- and 2nd-line treatment, as stated in current European Society for Medical Oncology and NCCN guidelines [7,8], although our results show the potential for FMT in earlier line as well. We also recommend using healthy donors from established fecal biobank which follow strict screening processes. Furthermore, an emerging clinical trial by Wang et al. recently released preliminary results showing strong evidence that FMT may be a safe and effective first-line treatment option for IMC, with 9 out of 12 patients achieving complete response [14], although more research is needed before routine application in this line. Despite unanswered questions, FMT offers significant advantages for patients’ refractory to standard medical treatment, including low cost, easy application, and low toxicity [31].

## 4. Conclusions

This case series contributes to the growing body of evidence [10,11,12,13,26,27] that suggests that FMT can be a safe and successful both as second- and third-line treatment for refractory IMC following standardized procedure, rigorous donor screening and the use of established fecal biobanks. An additional FMT can also be successfully applied if antibiotic treatment is required following the first procedure.

## Figures and Tables

**Table 1:** Effects on fecal microbiota transplantation (FMT) on quality of life (QoL) using European Organisation for Research and Treatment of Cancer QoL questionnaire C30 (EORTC QLQ-C30).

	Patient 1		Patient 2	
Parameter	Before FMT	After FMT	Before FMT	After FMT
Function scores (lower values represent worse functioning)				
Physical functioning	26.7	93.3	40.0	66.7
Role functioning	0.0	0.0	0.0	66.7
Emotional functioning	66.7	100.0	58.3	100.0
Cognitive functioning	100.0	100.0	83.3	66.7
Social functioning	33.3	66.7	66.7	100.0
Global Health	41.7	66.7	0.0	83.3
Symptom scores (lower values represent more significant symptoms)				
Fatigue	55.6	0.0	77.8	55.6
Nausea	0.0	0.0	0.0	0.0
Pain	100.0	0.0	55.6	0.0
Dyspnea	33.3	0.0	100.0	33.3
Insomnia	0.0	0.0	100.0	33.3
Appetite loss	100.0	0.0	0.0	0.0
Constipation	0.0	0.0	0.0	0.0
Diarhhoea	100.0	0.0	100.0	0.0
Functional difficulties	0.0	0.0	66.7	0.0

## Data Availability

The original contributions presented in this study are included in the article/Supplementary Material. Further inquiries can be directed to the corresponding author.

## Supplementary Materials

The following supporting information can be downloaded at https://www.mdpi.com/article/10.3390/ph18111719/s1: Supplementary Information of Exclusion criteria for donors include and Procedure for Fecal Microbiota Transfer (FMT) Treatment.
