# Supplementary material for: Fecal Microbiota Transplantation in Refractory Immune-Mediated Colitis: Case Series and Review of the Literature
Source: Pharmaceuticals (Basel). 2025 Nov 12;18(11):1719. doi: 10.3390/ph18111719 (PMC12655589; doi:10.3390/ph18111719)
Supplement: Supplementary file 1 [file pharmaceuticals-18-01719-s001.zip › pharmaceuticals-3929226-supplementary.pdf]

Exclusion criteria for donors include:

- Inflammatory bowel disease or other chronic gastrointestinal disorder (IBS, chronic diarrhea, or chronic constipation)
- History of malignant disease or current treatment with antineoplastic drugs
- Mental illness (depression, schizophrenia, autism, Asperger's syndrome)
- Chronic neurological/neurodegenerative disease (e.g., Parkinson's disease, multiple sclerosis)
- Autoimmune disease and/or use of immunosuppressants
- HIV, hepatitis A, B, C, or E, or known exposure within the past 12 months
- Chronic pain syndrome (e.g., fibromyalgia)
- Obesity (BMI > 30), metabolic syndrome
- Significant allergies (food allergens, multiple allergies)
- Recent gastrointestinal infection (within the past 6 months)
- Travel to countries with low hygiene standards, i.e., high risk for endemic diarrhea or possible infection with multidrug-resistant bacteria within the past 6 months
- Tattooing or body piercing within the past 6 months
- Promiscuous behavior
- Drug abuse
- Use of antibiotics within the past 3 months
- Use of other medications that may potentially affect the composition of the microbiome (e.g., proton pump inhibitors)

Procedure for Fecal Microbiota Transfer (FMT) Treatment

Fecal microbiota transfer (FMT) involves preparing the patient (cleansing of the large intestine with a standard protocol), after which stool from a healthy donor—previously tested according to current guidelines for infectious diseases and exclusion criteria—is processed, usually with saline solution at a ratio of 1:3–5. The homogenized preparation (stool solution) in a volume of 200–500 mL is then instilled into the recipient's intestine (during colonoscopy) or via a tube inserted through the nose into the stomach, duodenum, or further into the small intestine (jejunum). Both methods can also be combined.

The donor stool sample is processed in a microbiological safety cabinet. The weight of the delivered sample is determined on a precision laboratory scale. Fresh stool (50 g) should be diluted with sterile saline solution (150–200 ml) and homogenized in a blender. The stool suspension is filtered through a sterilized sieve and several layers of sterile gauze to remove all large particles. The resulting fecal filtrate is stored in a sterile container and can be used immediately for the FMT procedure (without the addition of glycerol). Instillation may involve freshly prepared donor stool suspension within 6 hours of collection or freshly frozen suspension thawed on the day of transfer.

If the sample is not immediately required for use, the sample can be transferred into sterile containers with screw caps, and sterile glycerol is added to a final concentration of 10%, clearly labeled, and frozen at -80 ºC. A sample prepared in this way can be stored for up to 2 years. When the sample is required for use, it is thawed gradually at room temperature or in a water bath at 30–37 ºC for 4–5 hours. The thawed sample should be used within 2 hours to prevent a decrease in the number of microbial cells.

Side effects of FMT are rare, but nausea, diarrhea, fever, or signs of systemic inflammatory response may occur. Worsening of intestinal inflammation may also rarely appear. In case of administration via nasogastric or nasoduodenal tube (i.e., application into the upper digestive tract), aspiration may occur in addition to the aforementioned side effects, and fatal outcomes due to aspiration of fecal material have been reported. All side effects that can otherwise occur during colonoscopy may also occur.

Before the application patient must have been informed, and must consent verbally and in writing, for both colonoscopy application and treatment with FMT. The benefits of the procedure and possible side effects of treatment have been explained to the patients beforehand.
